# Development of a Double-Gauss Lens Based Setup for Optoacoustic Applications

**DOI:** 10.3390/s17030496

**Published:** 2017-03-03

**Authors:** Hojong Choi, Jae-Myung Ryu, Jung-Yeol Yeom

**Affiliations:** 1 Department of Medical IT Convergence Engineering, Kumoh National Institute of Technology, Gumi 39253, Korea; hojongch@kumoh.ac.kr; 2 Department of Optical System Engineering, Kumoh National Institute of Technology, Gumi 39253, Korea; 3 School of Biomedical Engineering, Korea University, Seoul 02841, Korea

**Keywords:** optoacoustic applications, double-Gauss lens, transducer

## Abstract

In optoacoustic (photoacoustic) systems, different echo signal intensities such as amplitudes, center frequencies, and bandwidths need to be compensated by utilizing variable gain or time-gain compensation amplifiers. However, such electronic components can increase system complexities and signal noise levels. In this paper, we introduce a double-Gauss lens to generate a large field of view with uniform light intensity due to the low chromatic aberrations of the lens, thus obtaining uniform echo signal intensities across the field of view of the optoacoustic system. In order to validate the uniformity of the echo signal intensities in the system, an in-house transducer was placed at various positions above a tissue sample and echo signals were measured and compared with each other. The custom designed double-Gauss lens demonstrated negligible light intensity variation (±1.5%) across the illumination field of view (~2 cm diameter). When the transducer was used to measure echo signal from an eye of a bigeye tuna within a range of ±1 cm, the peak-to-peak amplitude, center frequency, and their −6 dB bandwidth variations were less than 2 mV, 1 MHz, and 6%, respectively. The custom designed double-Gauss lens can provide uniform light beam across a wide area while generating insignificant echo signal variations, and thus can lower the burden of the receiving electronics or signal processing in the optoacoustic system.

## 1. Introduction

Ultrasound is widely used in a variety of the applications such as skin, intravascular, and small animal imaging; nondestructive testing; and sound navigation and ranging, because ultrasound machines can provide safe, real-time information through non-ionizing radiation-based methods [1,2,3,4]. In contrast to X-ray and optical imaging modalities, ultrasound is able to achieve higher spatial resolution of a target deep within tissue but it often suffers from low contrast due to similar acoustic properties of soft tissues [5,6,7]. In other words, when array ultrasound transducers are used in a system, the echo signal quality of a target—e.g., a blood vessel or a tissue—is also affected by the speckle patterns and grating lobes, thus providing a low quality of the contrast ratio of the targets [8,9]. 

Recently, optoacoustic systems have been introduced because the low contrast of traditional ultrasound methods are unable to provide high enough resolution for soft tissues such as blood vessel and hemoglobin [10,11]. The optoacoustic systems can achieve a high optical contrast at ultrasound penetration depth and spatial resolution which are essential characteristics to differentiate soft tissue properties [12,13]. This is possible because, in the optoacoustic systems, the light is utilized as the transmitting source and a transducer as the receiving source to detect the acoustic signal [10]. However, the penetration depth is relatively lower compared to ultrasound-only systems because the light is typically diffused in the surface of the tissue and signal intensity of the echo signal in the optoacoustic systems are compromised in the process [14]. In order to increase the light intensity, the pulse repetition rate of the control signal for transmitted light has to be greater than that in the ultrasound-only method [15]. The configuration of transmission components and optimization of the light beam intensities are currently one of the technical issues to address in optoacoustic systems. 

In optoacoustic systems, lenses have been used to diverge or focus the lights. A concave lens usually diverges a beam in order to increase the field of the light, and the light is directly transmitted to a target such that a transducer has to be located perpendicularly to the target [16]. A conical lens can twist and focus a light beam to a certain location such that a transducer has to be located in the same line [17,18]. In optoacoustic systems, these types of the lenses are used in order to visualize the target with transducers. In order to scan the target in a large field of view, the transducers may translate from one position to another. The echo signals obtained from a certain area would not be uniform due to the non-uniform beam intensity as formed by the lens. When the received echo signals have varying amplitudes, variable-gain amplifier or time-gain compensation amplifier has to be used to equalize the echo signal amplitudes and form the target information [19]. In this study, we introduce a double-Gauss lens that can manipulate a light beam from a source, thus providing a large field of view with uniform light intensity [20,21]. To the best of our knowledge, we were the first to implement optoacoustic applications using a double-Gauss lens.

The efficiency of the lens basically needs to be as high as possible in order to irradiate high-intensity light on a sample or an object [22]. Therefore, one needs to design a high numerical aperture (NA) lens that satisfies this requirement. A high NA lens leads to a high optical efficiency of lens [22]. Generally, the lens design can be easier if the F-number is large and the field of the view of the lens is small [23]. Additionally, the lens size would be unnecessarily large if its focal length is too long [23]. Therefore, the lenses are preferably designed using a standard lens having an angle of view of 20–25°. Meanwhile, these type of lenses have been developed as a floating type or digital single lens reflex (DSLR)-type systems, where more than two lens groups should move [24]. Recently, compact system camera (CSC) systems without a mirror box in order to reduce the weight and size of the camera have been released [25]. However, they cannot use contrast auto focus (AF) systems such that the speed of the CSC systems is low compared to that of the DSLR-type systems [26]. In order to resolve this issue, entire lenses have to be designed to utilize AF using only one lens; we call it an inner focus-type lens. Therefore, we need to design an inner focus-type standard lens with a high f-number (F/#), in which only a single lens moves. 

**Figure 1:**
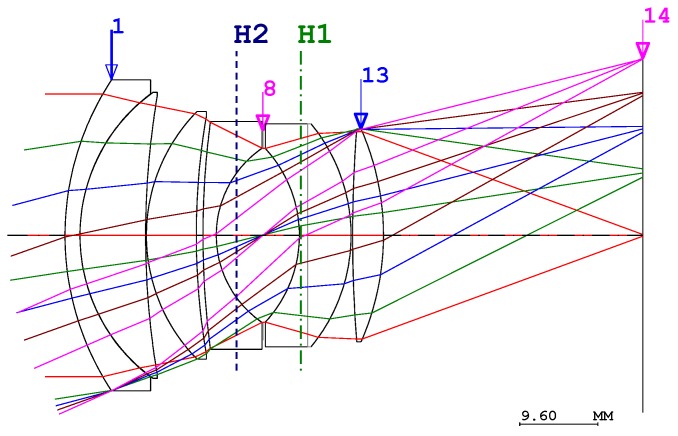
Optical layout of the conventional double-Gauss lens.

It is more challenging to design entire lenses in which a single lens moves to provide AF than a lens where more than two groups of lenses move to provide the same function [27]. In order to resolve this issue, we need to begin the design from the paraxial approximation. If the image plane size is similar to a 35 mm film, the angle of the view of the lens with a focal length of 50 mm is 23.4°. Therefore, we need to start the design of a double-Gauss-type lens with a focal length of 50 mm as illustrated in Figure 1. This example is obtained from the optical design tool (CodeV, Synopsys, Mountain View, Santa Clara, CA, USA).

## 2. Materials and Methods 

In order to design an inner focus-type standard lens in which a single lens moves to provide AF in the double-Gauss lens, it is favorable for the lens group to be positioned in advance in order to compensate residual aberrations [28]. Therefore, the double-Gauss lens uses one lens group, followed by a single AF lens and an additional lens group as shown in Figure 1. 

In all, the double-Gauss lens has three lens groups. This lens type can be described by the refractive power and the ray height from the upper side using the Gaussian bracket in Equation (1) [29]

(1) $$\begin{array}{l} \left[k_{1},-z_{1},k_{2},-z_{2},k_{3}\right]=K\\ \left[k_{1},-z_{1},k_{2},-z_{2},k_{3},-z_{3}\right]=0 \end{array}$$

where *k_i_* the refractive power of each lens group, *z_i_* is the distance between the principal planes of adjacent lens group, and *K* is the total refractive power of the entire lens. To solve Equation (1), variables other than *z_1_* and *z_3_* should have initial values. 

**Figure 2:**
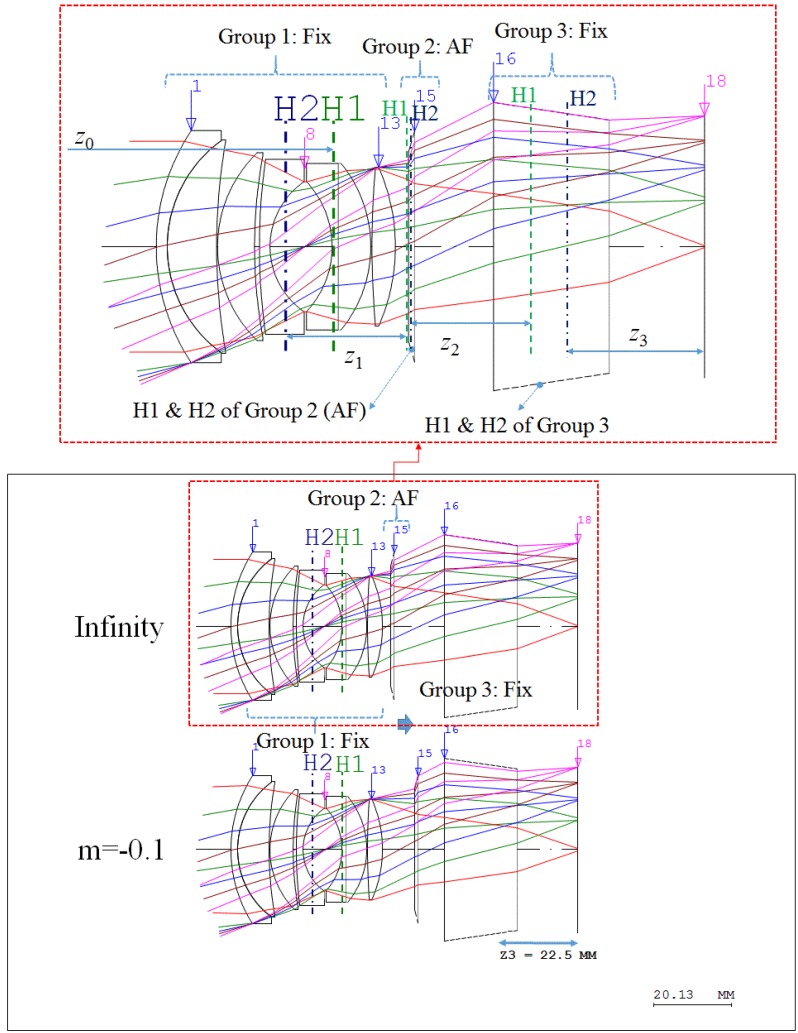
Optical layout of the paraxial design.

As shown in Figure 2, we can assume that the refractive power of Group 1 *k*_1_ is equal to *K* in Equation (1) if the total focal distance is 50 mm, even though the AF lens (Group 2) and the additional lens (Group 3) are included in the lens. Additionally, the lens should have appropriate air space between each lens group before and after the AF lens. This air space is to allow lens movement for focusing and also to compensate for variations in the image plane arising from manufacturing error of the double-Gauss lens. The first principal plane (H1) of the first lens group of the lens is shown, and the second principle plane (H2) of the first lens group is at a distance away from the image plane (surface 18), as shown in Figure 1. Therefore, the variable *z*_1_ can be equal to *z*_2_ to maintain the proper distance between the lens groups before and after the AF lens. The distance between the second principal plane (H2) and the last surface (surface 13) of the first lens group is 18.011 mm. Therefore, we need to assume that the variables *z*_1_ and *z*_2_ are equal to 20 mm. The distance *z*_3_ between the principal plane of the third lens group (Group 3) and the image plane (surface 18) is calculated to be 22.5 mm, as shown in Figure 2. The refractive power *k*_2_ of the AF lens and the refractive power *k*_3_ of the third lens group (Group 3) are unknown. Therefore, Equation (1) can be changed into Equation (2) according to these conditions.

(2) $$\begin{array}{l} \left[K,-z_{1},k_{2},-z_{1},k_{3}\right]=K\\ \left[K,-z_{1},k_{2},-z_{1},k_{3},-z_{3}\right]=0 \end{array}$$

Using the Gaussian bracket properties, the refractive power *k*_2_ of the AF lens and the refractive power *k*_3_ of the third group in Equation (2) can be converted into Equation (3)

(3) $$\begin{array}{l} k_{2}=\frac{1-Kz_{3}-2Kz_{1}}{z_{1}\left(1-z_{1}K\right)}=\frac{F-z_{3}-2z_{1}}{z_{1}\left(F-z_{1}\right)}\\ k_{3}=\frac{k_{2}\left(-1+z_{1}K\right)}{Kz_{3}}=\frac{z_{3}+2z_{1}-F}{z_{1}z_{3}} \end{array}$$

Here, *F* is the effective focal length of the entire lens, and it is the reciprocal to the refractive power. Therefore, we can calculate that *F* = 50.0 mm, *k*_2_ = −1/48 mm^−1^, and *k*_3_ = 1/36 mm^−1^. If we assume that the AF lens and the lens of the third group are perfect lenses without chromatic aberrations, we can obtain the lens after the power arrangement process is completed, as shown in Figure 2. The process to determine the refractive power of each lens group is called power arrangement. In Figure 2, we can classify two different cases: infinity and m = −0.1. In these cases, the distance from the object is infinite and the magnification of the lens (m) is −0.1. Equation (4) can be derived if the magnification of the lens (m) is given to calculate the moving distance of the AF lens (∆*z*).

(4) $$\begin{array}{l} \left[-z_{0},k_{1},-z_{1}+\Delta z,k_{2},-z_{2}-\Delta z,k_{3}\right]=\frac{1}{m}\\ \left[-z_{0},k_{1},-z_{1}+\Delta z,k_{2},-z_{2}-\Delta z,k_{3},-z_{3}\right]=0 \end{array}$$

where *z*_0_ represents the distance between the object and the first principal plane (H1) of the first lens group.

If the distances between the refractive power *k*_1_, *k*_2_, and *k*_3_ of each group and the principal planes (*z*_1_, *z*_2_, and *z*_3_) are infinite, there are two unknown variables in Equation (4), which are the moving distance of the AF lens (∆*z*) and the distance between the object and the first principal plane of the first lens group (*z*_0_). Therefore, we can obtain ∆*z* to be 6.16 mm in Equation (4) using a MATLAB program (MathWorks, Natick, MA, USA). The AF lens and the third lens group, being aberration-free and thin lens systems, must be converted to real lenses. As the AF lens has to be physically lightweight, only one AF lens and two lenses in the third lens group (Group 3) must be used to increase the moving velocity of the AF lens. In Equation (5), we can obtain the form factor of the lens (X) if the given spherical aberration is minimized. The spherical aberration *S_I_* is not zero for a single-type lenses [22]. The variable *h* represents the height of the axial ray at the lens front surface; *k*_2_ represents the refractive power of the AF lens; variable *n* represents the refractive index of the AF lens material itself; *m*_2_ represents the AF lens magnification; valuable *c*_1_ represents the curvature values of the lens front surface, and *c*_2_ represents the curvature values of the lens rear surface.

(5) $$\begin{array}{l} S_{I}=\frac{1}{4}h^{4}k_{2}^{3}\cdot \left\{\frac{n+2}{n{\left(n-1\right)}^{2}}{\left(X+\frac{2\left(n^{2}-1\right)}{n+2}Y\right)}^{2}+{\left(\frac{n}{n-1}\right)}^{2}-\frac{n}{n+2}Y\right\}\\ X\equiv \frac{c_{1}+c_{2}}{c_{1}-c_{2}},Y\equiv \frac{1+m_{2}}{1-m_{2}} \end{array}$$

If the refractive power is given when the lens thickness is not considered in Equations (5) and (6), the curvature of the front and rear surfaces *c*_1_ and *c*_2_ of the AF lens can be calculated [30].

(6) $$k_{2}=\left(c_{1}+c_{2}\right)\left(n-1\right)$$

**Figure 3:**
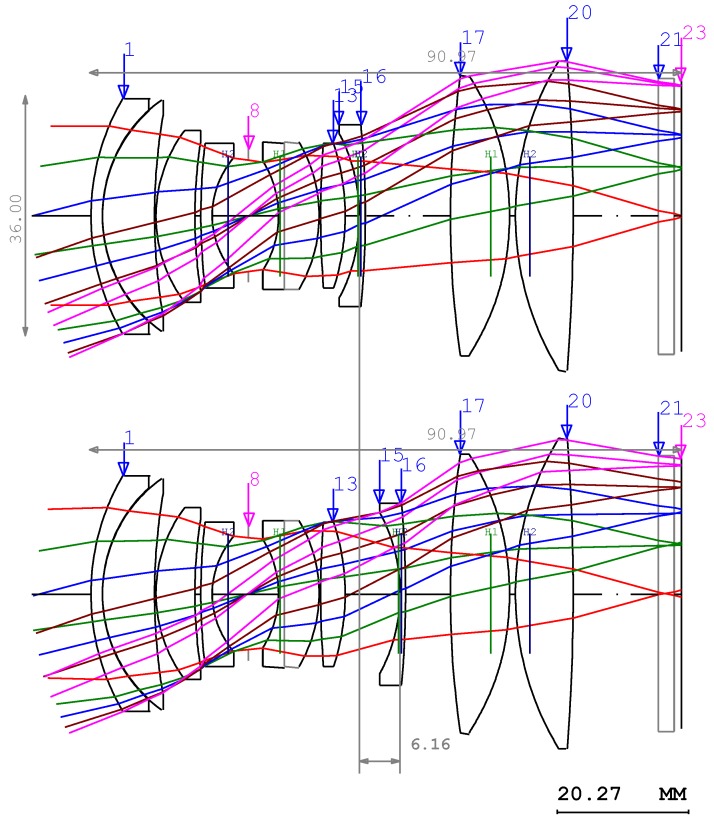
Optical layout of the designed double-Gauss lens.

For the two lenses in the third lens group, the lens module design method should be used unlike in Equation (5) [31]. Using this method, the initial layout of the lens can be determined as shown in Figure 3. As shown in Figure 2, the added Group 2 and Group 3 have negative power and positive power, respectively. Group 2, used for focusing function, is comprised of only one lens while Group 3 is comprised of two lenses. The shape of each lens was determined to minimize Seidel aberration and each lens material was selected to eliminate chromatic aberration [32]. The lens with completed power arrangement is, thus, constructed as shown in Figure 3.

Here, flint glass was used for the lens in Group 2 with negative power and crown glass was used for the lenses in Group 3 with positive power to minimize the color aberration of the entire system [33]. However, the performance of the entire lens is still low even though Seidel aberration was corrected. Therefore, we should resolve the non-linear simultaneous equations about the curvature, thickness, and refractive index of the lens material in order to minimize residual aberrations. The process to solve these complex non-linear equations is called the optimization [34]. In Figure 3, the designed double-Gauss lens should be compensated for chromatic aberration such that we can design and optimize a high-performance lens with minimal aberration, thus producing uniform light beam intensity in the area of interest. 

## 3. Results

### 3.1. Double-Gauss Lens Fabrication

**Figure 4:**
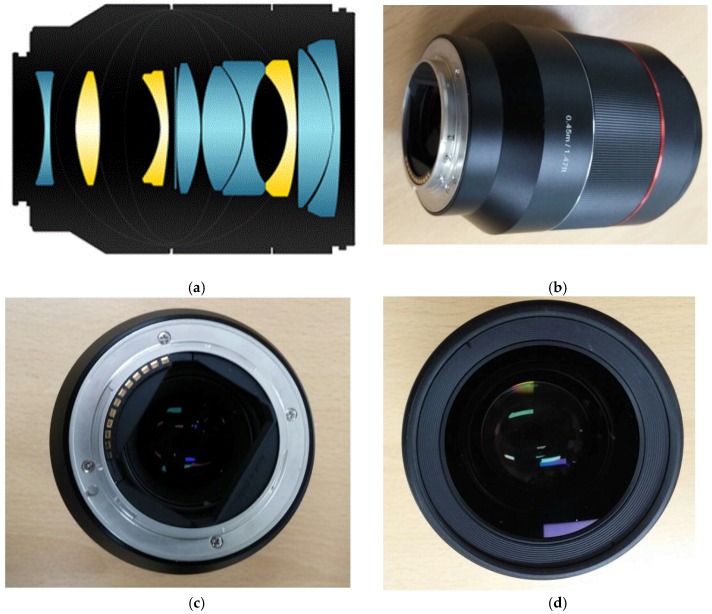
(**a**) Interior and (**b**) exterior images of our fabricated double-Gauss lens; (**c**) top and (**d**) bottom view image of our manufactured double-Gauss lens.

Figure 4 illustrates the interior and exterior structures of the manufactured product after the performance optimization process. Upon performing performance optimization, the lens structure was modified, and nine lenses were utilized to construct the double-Gauss lens. The original design (Figure 3) was a biconvex lens. However, it was changed into a biconcave lens because the final lens should be a field flattener-type lens to compensate for the field curvature of the double-Gauss lens.

**Figure 5:**
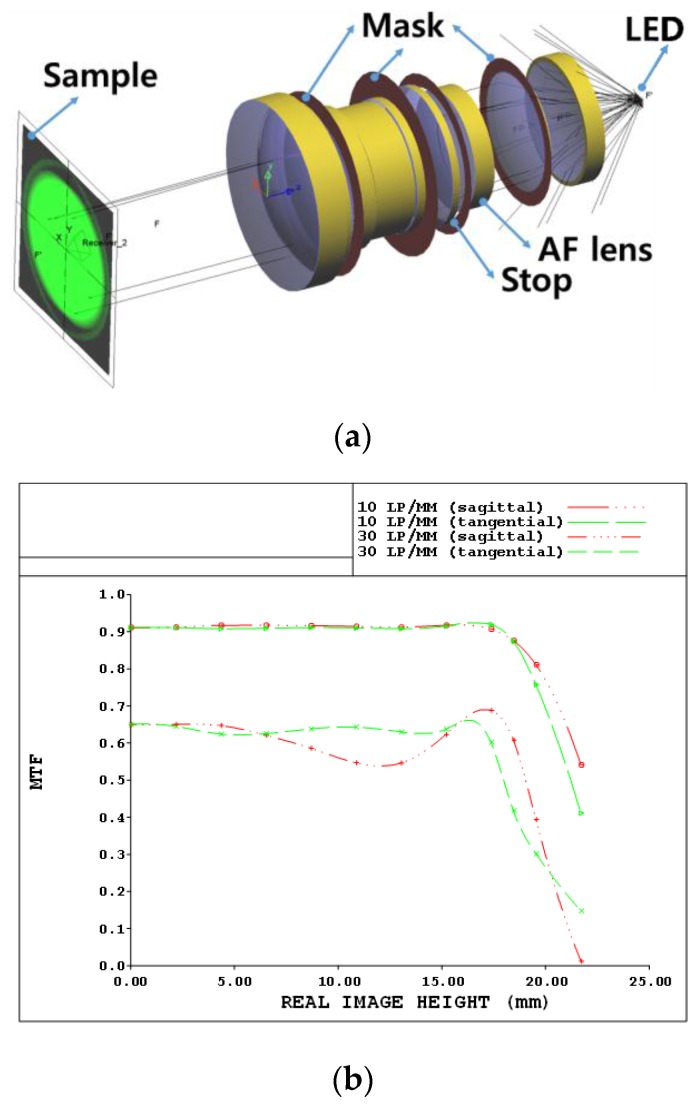
(**a**) The three-dimensional optical layout; and (**b**) the MTF graph of the designed double-Gauss lens.

As shown in Figure 4, the designed double-Gauss lens was manufactured to achieve sufficient performances for the modulation transfer function (MTF) [35]. In Figure 5a, the three-dimensional optical layout of the designed double-Gauss lens is illustrated. In addition, we provide the expected modulation transfer function (MTF) graph to verify the resolving power performance of the lens. The image formation capability of our designed double-Gauss lens using CodeV software was confirmed as follows. Figure 5b shows the MTF graph of the optimized lens where “lp/mm” represents the units of the spatial frequency as the number of line pairs per 1 mm. Thus, 10 lp/mm means that there are 10 pairs of 0.05 mm thick black and white lines per 1 mm. “Sagittal” and “tangential” respectively imply that the lines are placed horizontally and vertically in an arbitrary cross section of the lens. As shown in Figure 5, there is no resolution degradation with the designed double-Gauss lens system up to 15 mm image height due to acceptable MTF variances within this range.

In this work, we used light modulation for the optoacoustic applications such that after the light was located in the image plane of the designed double-Gauss lens, we investigated the distribution of the light intensity in the opposite plane. Therefore, the distribution of the light intensity is expected to be uniform. 

A sample and an LED were located at the left and right positions, respectively. The light was passed through the lens and irradiated on the sample. A light emitting diode (LED) light source with its wavelength in the visible ranges is used as the transmit source in the developed optoacoustic systems because LED lights in the visible wavelength and power range used in this study are innocuous. However, there may be some errors—such as the location of light and the distance between the lens and the object—when this lens is utilized in optoacoustic systems. Therefore, we should verify the distribution of the LED light intensity with respect to the aberration caused by the location errors and the distances between the double-Gauss lens and the object in an optoacoustic system as the AF lens in the double-Gauss lens should move to provide AF.

### 3.2. Double-Gauss Lens Performance Verification

**Figure 6:**
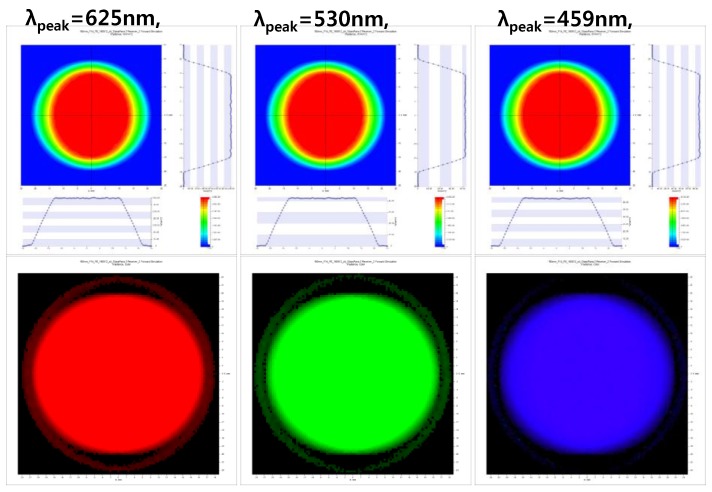
The light intensity distribution profiles on the sample with the red, green, and blue LEDs.

Figure 6 shows the light intensity distribution profiles at distances of 60 mm using red, green, and blue LEDs with peak wavelengths of 625, 530, and 459 nm, respectively. The full width half-maximum (FWHM) values of the red, green, and blue LEDs were 16, 35, and 22 nm, respectively. The powers of the red, green, and blue LEDs were 0.29, 0.36, and 0.60 W, respectively, because the efficiency of the LEDs is dependent on their LED wavelengths. Additionally, the intensity profiles in the horizontal and vertical planes were slightly different because the LEDs were rectangular in shape.

**Figure 7:**
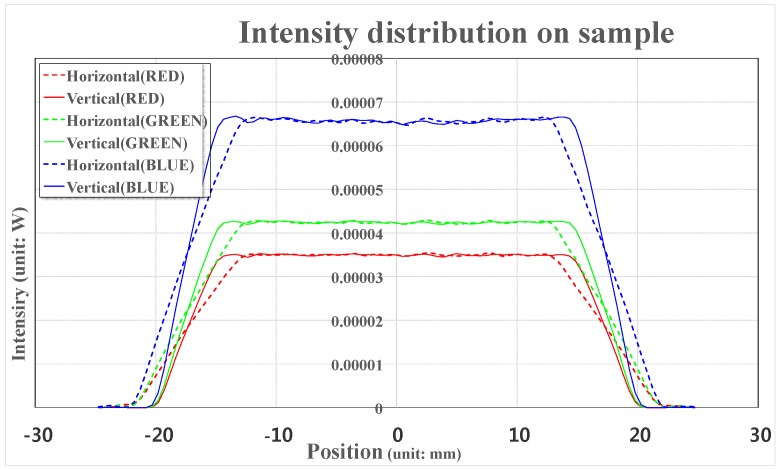
Light intensity distributions of the red, green, and blue LEDs with wavelengths of 628, 524, and 460 nm, respectively.

The light intensity distribution profiles of the LEDs were calculated in Figure 7. As shown in Figure 7, the light intensity distributions are almost constant and the light intensities of the red, green, and blue LEDs at the central area are 0.035, 0.042, and 0.065 mW, respectively.

**Figure 8:**
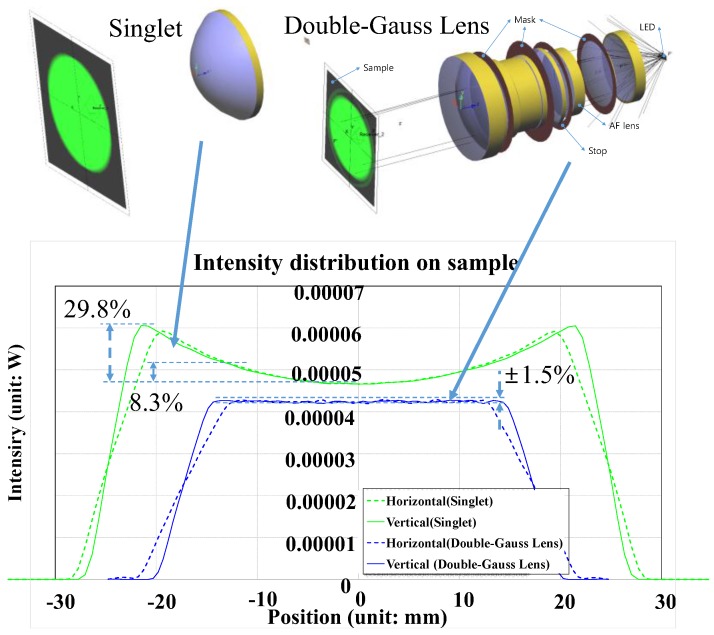
Optical layout and light intensity distributions of the single lens and our designed lens.

Figure 8 illustrates the optical layouts and light intensity distributions of a commonly obtainable 50 mm diameter single lens (67–277, Edmund Optics, Barrington, NJ, USA) and the custom designed lens. The graph shows that the light intensity distribution of a single lens (singlet) changes with position (29.8%) while that of our custom designed lens show negligible change (±1.5%). This is because the aberration of a single lens was not fully corrected, even for the one with an aspherical surface. 

The light intensity distribution profiles were obtained under the assumption that the double-Gauss lens was constructed without any chromatic aberrations. Due to manufacturing errors that can manifest themselves during experiments, light intensity distribution variability caused by chromatic aberrations should be taken into account in the experiment. Moreover, positioning errors of the AF lens can arise when mirror-less interchangeable lens modules are separable from the camera body after the power of the camera body is turned off. However, we used the double-Gauss lens without a camera body, and thus did not consider such effects.

**Figure 9:**
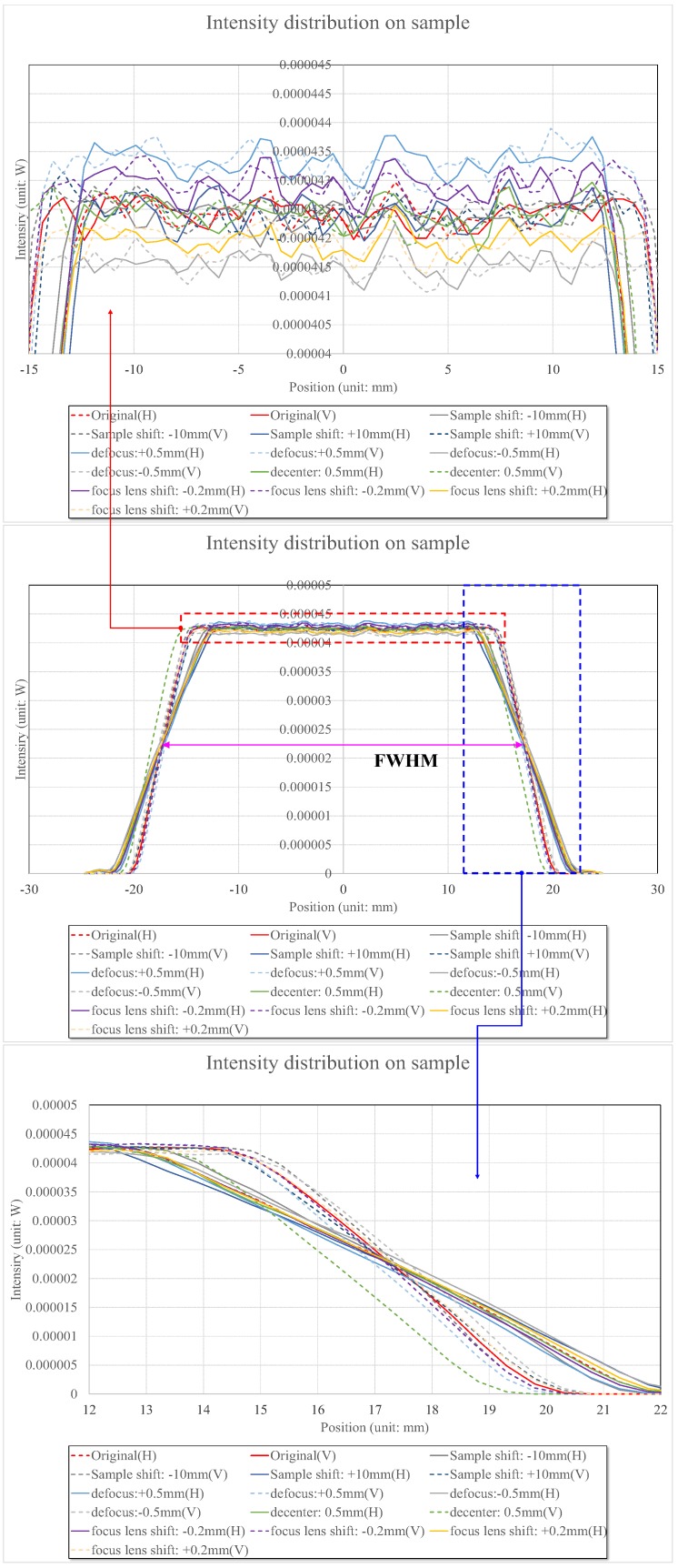
Intensity profile on sample by lens error.

Figure 9 illustrates the cross-sectional diagrams of the light intensity distribution profiles of the green light with a wavelength of 530 nm caused by the types of errors mentioned above. In Figure 9, we call it ‘defocus’ when the LED location errors are generated in the front and rear directions of the optical axis, ‘decenter’ when the LED location errors are generated in the top and bottom directions of the optical axis, ‘shift’ when LED location errors are generated in the front and rear directions of the sample location, and ‘focus lens shift’ when the AF lens errors are generated in the front and rear directions. The results when red, green, and blue LEDs are used can similarly be obtained because the designed double-Gauss lens in our research was thoroughly compensated for color aberration. Figure 9 represents the light intensity distributions caused by the defocus, which exhibits errors along the optical axis. When the sample shifted 10 mm, or the defocus and the decenter moved 0.5 mm, or focus lens shifted 0.2 mm, the intensity values increased from 0.0416 mW to 0.0433 mW between zero positions of −15 mm and +15 mm, and these values were 2.1% compared to those of lenses without any errors. Therefore, this implies that the light intensity distribution variations caused by color aberration or manufacturing error can be neglected in our optoacoustic system and should not affect system performance. As the FWHM, in addition to the light intensity, also remained constant, the physical quantity F/# or NA—which represents the index of the brightness of the lens—also changed slightly. Therefore, the lens was well designed with uniform light intensity for optoacoustic systems.

### 3.3. Pulse-Echo Response with Double-Gauss Lens

**Figure 10:**
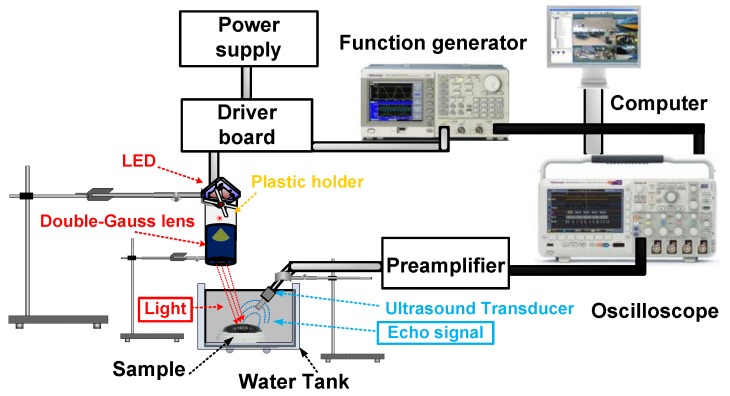
Experimental optoacoustic system with designed double-Gauss lens. The sample was placed in a tank filled with de-ionized water and water temperature remained constant throughout the experiments.

Figure 10 shows the experimental setup of the developed optoacoustic system using the double-Gauss lens. The A-mode pulse-echo response is one of the typical methods to evaluate an optoacoustic system when numerous components are integrated into the entire system. In the optoacoustic system, visible LED lights (CBT-120) whose wavelengths are between 400 nm and 700 nm were used on eye tissue samples of a bigeye tuna. 

A function generator (AFG3252, Tektronics Inc., Beaverton, OR, USA) generated a 1 kHz and 5V_p-p_ pulsed signal which controls the pulse mode in a driver board (DK-136M-1, Luminus Devices) in order to produce the pulsed LED light. The function generator was connected to an oscilloscope (MSO2024B, Tektronics) to synchronize the timing pulses. A plastic holder fabricated with polycarbonate material in a 3D printer (Fortus450mc, Stratasysis, Edina, MA, USA) was used to firmly hold the LED and the double-Gauss lens. The portion of diverging LED light that converged on the designed double-Gauss lens was focused onto the bigeye tuna tissue sample in a parallel direction. The focused light generates thermally excited ultrasound waves within the sample, and these echo waves were in turn detected by an in-house fabricated 10 MHz transducer with 2 mm focal distance. Signals were read out by a 20 dB voltage operational amplifier and active Sallen-Key low pass filter with 20 dB gain and 100 MHz −3 dB bandwidth in order to amplify the received echo waveforms. The echo waveforms were acquired by the oscilloscope (MSO2024B, Tecktronics), digitized and their spectrum in the frequency domain were plotted using MATLAB program (MathWorks, Natick, MA, USA) in a computer.

**Figure 11:**
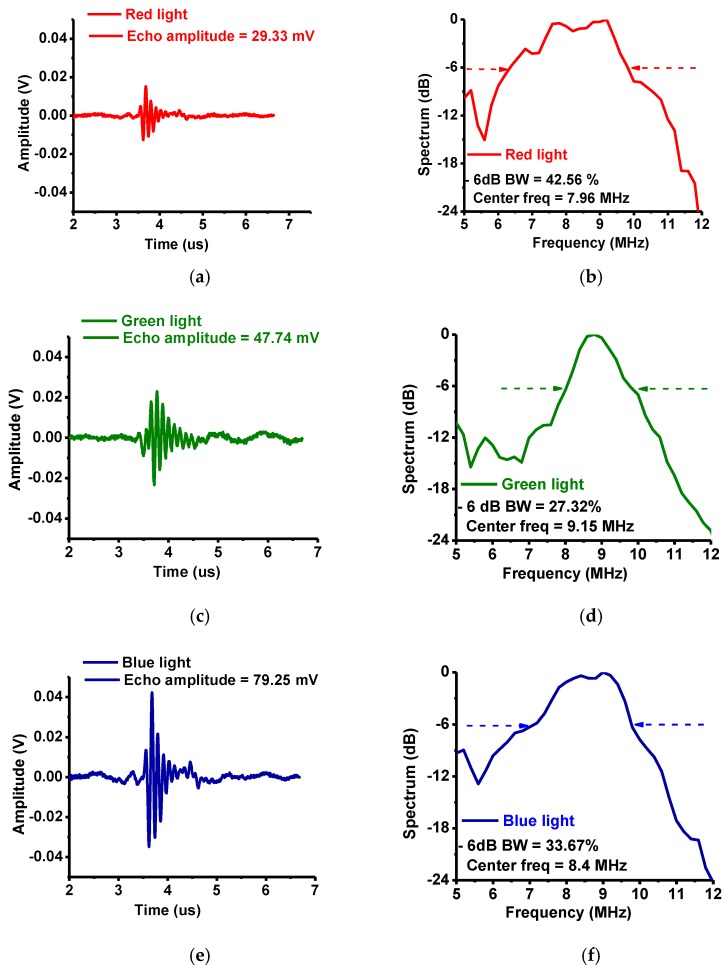
The echo waveform data of the developed system. (**a**) Echo amplitude and (**b**) its spectrum with red light, (**c**) echo amplitude and (**d**) its spectrum with green light, (**e**) echo amplitude and (**f**) its spectrum with blue light.

Figure 11 shows the echo waveforms in the time and frequency domains. When red, green, and blue lights were used, the detected echo waveform amplitudes were 29.33, 47.74, and 79.25 mV, respectively and the center frequencies were 7.96, 9.15, and 8.4 MHz with −6 dB bandwidths (percentages) of 42.56, 27.32, and 33.67%, respectively. Each echo waveform center frequency was obtained by averaging the frequencies at the measured −6 dB bandwidths as shown in Figure 11b,c,f. Slight shifts in the measured center frequencies of the echo waveforms may have been caused by the long coaxial cables (4 m) between the transducer and the preamplifier and attenuation in the layers of the tissue samples. However, we were able to acquire spectra of reasonable amplitudes with the setup. 

**Figure 12:**
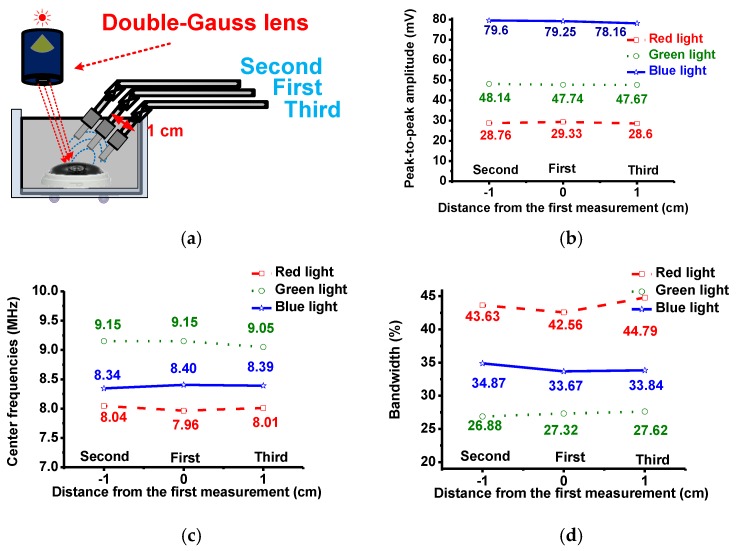
(**a**) Experimental setup when the transducers were placed diagonally in the right side and (**b**) peak-to-peak amplitudes; (**c**) center frequencies; and (**d**) −6 dB bandwidths of the echo signals.

**Figure 13:**
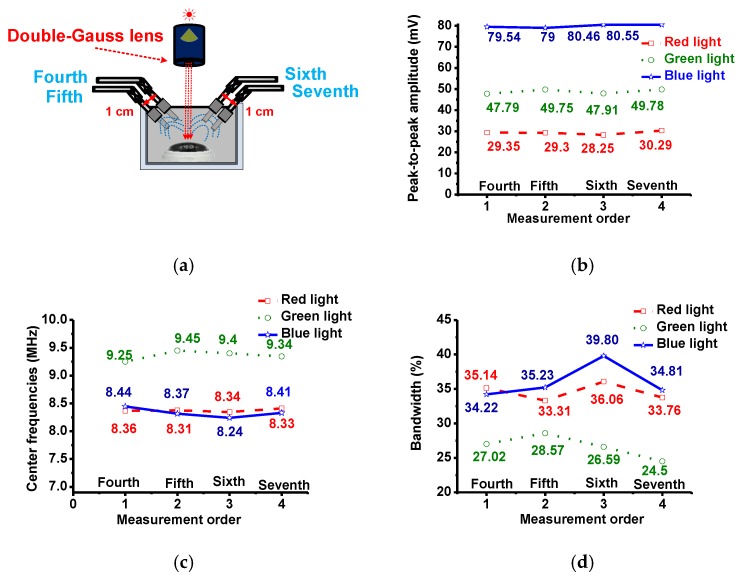
(**a**) Experimental setup when the transducers were placed in the left and right sides and (**b**) peak-to-peak amplitudes; (**c**) center frequencies; and (**d**) −6 dB bandwidths of the echo signal.

Figure 12a and Figure 13a show the different setup configurations of the LED light source, lens, and transducers to verify echo signal uniformity with position. As depicted in Figure 12a, in the ‘first’ measurement, a transducer was at an angle from the incident light such that the light was focused on one side of the sample and the reflected echo signals were detected from the other side. In the ‘second’ and ‘third’ measurements, the transducer was shifted 1 cm in both directions away from the first position to check for any echo signal variations associated with the incident light beam. Figure 12b,c,d show the measured peak-to-peak amplitudes, center frequencies and −6 dB bandwidths of the echo signals. Relative to each other, the calculated deviations of the peak-to-peak amplitudes, center frequencies and their −6 dB bandwidth variations of the echo signals in the second and third measurement data were less than 6%. These measurement data verified that the designed lens could produce a uniform beam, thus generating uniform echo signal data accordingly. 

In Figure 13, the light through the lens irradiates the samples perpendicularly and a transducer was placed at an angle from the normal. Measurements were taken from both sides of the normal plane. At each position (‘fourth’ and ‘sixth’ measurements), another measurement was made after a shift of 1 cm (‘fifth’ and ‘seventh’ measurements). Figure 13b–d show the measured peak-to-peak amplitudes, center frequencies and −6 dB bandwidths of the echo signals. In this setup, relative to each other, the calculated variations of the peak-to-peak amplitudes, center frequencies, and their −6 dB bandwidths variations of the echo signals in the second and third measurement data were also small—less than 2 mV, 1 MHz, and 6%, respectively. 

These experimental data confirmed that the developed optoacoustic system integrated with a double-Gauss lens could produce uniform light beam intensity with uniform echo signal across the illumination region. In the typical acoustic array system, the different echo signal amplitudes need to be adjusted by additional variable gain amplifiers or time-gain compensation amplifiers to improve the signal quality but in process also increase the noise level of the system as a whole [36,37]. Therefore, having uniform echo signal amplitude could help to lower the system and successive signal processing burden.

## 4. Conclusions 

We proposed an optoacoustic system using a double-Gauss lens with the goal of providing a large field of view with uniform light intensity. Such a configuration would reduce system burden associated with equalizing the detected echo signal amplitudes because currently developed lens configurations used in optoacoustic systems do not provide this kind of uniform beam intensity across a wide area. In order to evaluate the performance of our custom designed double-Gauss lens, we simulated the light intensity profiles across the illumination region of red, green, and blue LEDs (0.29, 0.36, and 0.60 W respectively). The intensity varied a mere 0.0416 mW to 0.0433 mW across a range of –15 mm to +15 mm. We also performed a pulse-echo test to assess the performance of the double-Gauss lens integrated optoacoustic system. With a 10 MHz transducer, the measured echo amplitudes were 29.33, 47.74, and 79.25 mV and their center frequencies were 7.96, 9.15, and 8.4 MHz, respectively for the three LEDs. Echo signals at different locations were also measured to verify the uniformity of the echo signal and the light source. Relative to the average of each measurement parameter, the echo signal peak-to-peak amplitude, center frequency, and −6 dB bandwidth variations were less than 2 mV, 1 MHz, and 6%, respectively. To conclude, these experimental data confirmed that we can use double-Gauss lens integrated optoacoustic systems for detecting tissue samples across ~2 cm distance with uniform echo signal intensity. This could help lower the burden of receiving electronics—such as the variable gain amplifier or time-gain compensation amplifier—in the system, and ease successive signal processing.
